# Follow My Eyes: The Gaze of Politicians Reflexively Captures the Gaze of Ingroup Voters

**DOI:** 10.1371/journal.pone.0025117

**Published:** 2011-09-21

**Authors:** Marco Tullio Liuzza, Valentina Cazzato, Michele Vecchione, Filippo Crostella, Gian Vittorio Caprara, Salvatore Maria Aglioti

**Affiliations:** 1 Department of Psychology, University of Rome “La Sapienza”, Rome, Italy; 2 IRCCS, Fondazione Santa Lucia, Rome, Italy; Kyushu University, Japan

## Abstract

Studies in human and non-human primates indicate that basic socio-cognitive operations are inherently linked to the power of gaze in capturing reflexively the attention of an observer. Although monkey studies indicate that the automatic tendency to follow the gaze of a conspecific is modulated by the leader-follower social status, evidence for such effects in humans is meager. Here, we used a gaze following paradigm where the directional gaze of right- or left-wing Italian political characters could influence the oculomotor behavior of ingroup or outgroup voters. We show that the gaze of Berlusconi, the right-wing leader currently dominating the Italian political landscape, potentiates and inhibits gaze following behavior in ingroup and outgroup voters, respectively. Importantly, the higher the perceived similarity in personality traits between voters and Berlusconi, the stronger the gaze interference effect. Thus, higher-order social variables such as political leadership and affiliation prepotently affect reflexive shifts of attention.

## Introduction

Possibly because of their unique morphology, human eyes are specially adept to mediate fundamental non-verbal communication skills [1]. In particular, gaze direction powerfully modulates social interactions at both explicit and implicit levels [2]. Detecting the gaze of other individuals reveals where they are attending [3], [4], [5], signals potential sources of reward or danger and activates basic motivational-emotional, approach-avoidance responses [6]. The reflexive shift of visuo-spatial attention is considered an early social cognitive ability leading to the later developmental ability to infer others' mental states [7], [8]. Social attention may rely upon a neural network where subcortical nodes mediate crude and largely unconscious, fast orienting responses to interpersonally relevant stimuli and cortical nodes subserve slower and conscious, context-dependent appreciation responses [9].

Gaze-mediated attentional capture is a fundamentally adaptive function that may be triggered automatically and thus be comparatively impervious to the influence of higher-order socio-cognitive variables. Tellingly, however, low- social status male rhesus macaques reflexively follow the gaze of any familiar rhesus macaques, but high-status macaques selectively follow the gaze of other high-status monkeys [10].

Social attention relies on gaze following behavior, the automatic tendency to imitate the oculomotor behavior of others [11], [12], [13], which is at the very basis of the development of other social cognitive skills [14]. This automatic imitative behavior seems to be subserved by a neural mirroring mechanism [15] similar to the one at play in during action observation in monkeys [16]. Relevant to the present study is that, albeit automatic, the motor resonance triggered by perception of others' actions seems to be modulated by the similarity between the observer and the model [17], [18].

Humans have developed large-scale political behavior, a very complex form of social behavior that requires an even more complex form of social knowledge and cognition [19]. Evidence of simpler political behavior in chimpanzees [20] and capuchins [21] suggests that we may have evolved in ways that maximize our capabilities for small-scale interactions. At least in western societies people are involved in multiple political activities, participate to elections, and join political groups. Choosing a party or a political group generically gives us a social identity [22]. Affiliation allows us to categorize rapidly and effortlessly other individuals as in-group and out-group. This act of categorization may be made consciously or unconsciously [23]. In fact, categorization of people into in-groups and out-groups has been observed after just milliseconds of mere exposure to persons or ideas about persons, with little or no effort, intention, awareness, or conscious control [24].

Beyond the mere affiliation, political ideology seems to have important social psychological functions [25]. Jost and colleagues [26], for instance, have described political conservatorism as a form of motivated social cognition which includes personality traits as authoritarianism [27] and ideological rationalizations as social dominance orientation [28]. These psychological differences between ideologies have been found to be reflected also in general neurocognitive functions [29] and in tasks where the sensitivity of the attention to social signals as the gaze of a schematic character [30] is tested. Furthermore, research on the moral foundations of ideology [31] has shown that conservatives, compared to liberals, endorse more moral values as loyalty to authority and to their own group.

Here we expand monkey studies [10] by investigating in humans whether reflexive attention might be influenced by social identity variables. To this aim, we explored whether reflexive gaze shifts were influenced by political affiliation, a process that allows conscious or unconscious, rapid categorization of individuals as in-group or out-group [23].

We tested 28 participants who were assigned to a left-wing (N = 15) or right-wing (N = 13) group on the basis of a questionnaire assessing their political orientation and voting behavior. Participants were required to perform a saccade towards a left- or a right-sided black square (target) when a black central square turned into red or blue respectively (imperative, instruction signal). The black square was positioned between the eyes of a political character face gazing straight to the participant. 75 milliseconds before the imperative central square color change, the character made a left- or right-ward saccade, either congruent or incongruent with the direction cued by the imperative signal. The character faces used in the present study, portrayed well-known, current or former political leaders and opinion makers, in order to disentangle the possible modulating role of the actual influence on the political landscape and/or the mediatic exposure. For these reasons we chose the pictures of the following personalities: Silvio Berlusconi (the most important centre-right wing, current prime-minister, political leader), Bruno Vespa (centre-right wing, opinion maker), Antonio Di Pietro (centre-left wing, current political leader) and Romano Prodi (centre-left wing, former prime-minister, no longer active as political leader). We used the difference in the accuracy between congruent and incongruent trials as an index of the interference of the models' gaze on the onlookers' oculomotor response.

To explore whether the influence of political affiliation on reflexive gaze following is linked to dispositional factors (e.g. the perceived similarity between oneself and specific political characters), we capitalized on social psychology studies emphasizing the relationship between the voters' personality characteristics and their political affiliation [32]. We focused on a conceptual framework that highlights the similarity between personality traits of voters and of same- or different-affiliation political leaders. In particular, we predicted that higher perceived similarity with a politician induced stronger gaze following behavior in a voter. Participants rated how much each item in a list of 25 adjectives representative of each dimension of the Big Five [33], [34] described themselves and four different political characters. Differences between the ratings concerning self (the voter) and others (each of four characters) provided a measure of the perceived similarity between voters and politicians.

## Materials and Methods

### Ethics statement

The experimental procedures were approved by the Fondazione Santa Lucia Ethics Committee (14/05/2008) and were carried out in accordance with the principles of the 1964 Declaration of Helsinki.

### Participants

Twenty-eight subjects (12 males, mean age = 25.25; SD = 2.89) gave their written informed consent to participate in the study. All had normal or corrected to normal vision with no history of neurological or psychiatric disease and were naïve to the purposes of the study. On the basis of a questionnaire assessing political preference and voting behavior (see below for more details), 15 participants were assigned to the left wing (9 females) and 13 subjects to the right wing group (7 females). The two groups were matched in age (*t*(26) = .03, *p* = .97), education (*t*(26) = .38, *p* = .70) and interest in politics (*t*(26) = 1.59, *p* = .12).

### Stimuli and Procedures

#### Eye movement recording

The study was performed in a quiet room with medium illumination (about 64 cd/m2). Subjects sat on a comfortable chair in front of an LCD monitor, positioned at about 57 cm from their eyes. Eye position and eye movements were measured monocularly in real-time by means of an infrared video-based system (ASL 504 Remote Tracker, Applied Science Laboratories, USA). The experiment was created with E-Prime software (version 1.1, Psychology Software Tools, Inc., Pittsburgh, PA) running on an IBM compatible computer. Each trial started with the appearance of a black central fixation square (0.21°×0.21° in size) presented on a light gray (about 47 cd/m2) background, and of two larger black squares (0.43°×0.43°) presented at 10.2° of eccentricity in the left and the right visual field. The fixation square was presented on the between-eyes point of the face of a political character with straight gaze. After 575 ms, the color of the central square changed to either blue or red). This was the imperative signal for the participants to make a fast and accurate saccade toward the left (change into blue) or the right (change into orange) target square. The colored cue remained visible until the end of the trial. 75 ms before the onset of the instruction-cue (stimulus onset asynchrony, SOA) the distracting character made a left- or right-ward saccadic movement. This interval was chosen because we demonstrated that gaze following behavior is maximal at this interval [11], [12], [13]. The characters used as distractors where: Antonio Di Pietro; Romano Prodi; Silvio Berlusconi; Bruno Vespa. It is also important that, at the data collection time (i.e. between 24th of July, 2009 and 24th October, 2009) the index of the trust in Berlusconi, varied between 55% (August 2009) and 60% (October 2009), as emerged by the “CRESPI Ricerche” phone CATI method survey (available at http://www.sondaggipoliticoelettorali.it/) on a 1,000 people sample stratified for sex, age, geographic area and population center size.

For each character-face we prepared a RGB digital photography (6.76°×6.76°). The original pictures were collected by searching in internet and modified by means of the Adobe Photoshop software (Adobe Systems Incorporated). To enhance their saliency, the stimuli were animated by two frames presented in rapid sequence. The first frame (lasting 500 ms) was replaced by a second frame lasting 875 milliseconds. The first frame depicted a straight gaze. The second frame depicted a gaze that could be oriented leftward or rightward. The direction of the character gaze and that one indicated by the instruction-cue could be congruent (e.g. both leftward) or incongruent (e.g. one leftward and the other rightward). Importantly, subjects were instructed to ignore the distracting stimulus and to focus their attention on the central square color change. Subjects were tested in four separate blocks, each associated with a character face. In each block, the two instruction cues (leftward or rightward) and the two distractors (congruent or incongruent) were equally probable and were presented in a random sequence. Each of the 4 possible combinations was equally probable and was repeated 12 times. Thus, a total of 48 trials per block was run. We analyzed the participants' directional accuracy by focusing on the first horizontal saccade that followed the instruction cue and had an amplitude larger than 2°. Saccadic RTs were also collected. Only RTs for correct trials were considered. The trials in which there was no clear evidence that a saccade occurred were excluded (725 out of 5376, 13.5%). A trial was rejected from the analysis described below if the latency was either less than 100 ms (anticipations) or greater than 500 ms (delays). The proportion of rejected trials was 3.4% of the total trials.

#### Measures of Voters' dispositions and personality

Participants filled out a self-report questionnaire in which the following measures were recorded: i) socio-demographic variables, as gender, age, and education level; ii) interest in politics as attested by frequency of discussion on the topic with their a) family members, b) colleagues at work, c) acquaintances, and d) friends (from 1 = “never” to 5 = “every day”). A single index of interest in politics was obtained by averaging the five ratings; iii) political orientation along a 7 point Likert like scale where 1 represents extreme left wing, 4 center, and 7 extreme right wing; iv) voting behavior in the last European political elections (June, 2009).

Participants were also shown the face of each character and asked to rate (along five-point Likert scales) the following:

1. Exposure: “please rate how much do you know the political character and his personality where 1 is “I know him very well” and 5 is “I do not know him at all”;
2. Influence: “please rate how much do you think this character is influent within the Italian political landscape” where 1 is “very influent” and 5 is “not influent at all”;
3. Positive emotions: “please rate how much do you think this character evokes positive emotions” where 1 is “not positive at all” and 5 is “very positive”;
4. Negative emotions: “please rate how much do you think this character evokes negative emotions” where 1 is “not negative at all” and 5 is “very negative”;

#### Assessment of personality similarity between Voters and Characters Personality

Participants rated themselves and separately each the four political characters on the Five Factors of personality (Energy/Extraversion, Agreeableness, Conscientiousness, Emotional stability, Openness [33]) using a list of 25 adjectives [34]. The list included five markers of: Energy/Extraversion (happy, determined, dynamic, energetic, active); Agreeableness (cordial, generous, loyal, sincere, unselfish); Conscientiousness (efficient, scrupulous, precise, conscientious, diligent); Emotional stability (optimistic, self-confident, solid, relaxed, calm); and Intellect/Openness to experience (sharp, creative, innovative, modern, informed). The adjectives were selected from a larger list of adjectives that have previously been identified in the Italian lexicon as being among the most frequently used to describe human personality and also the most representative of each of the dimensions of the Big Five. Each adjective was rated for how characteristic it was of each target on a 1 (“not at all”) to 5 (“very much so”) scale. We measured the perceived personality similarity in personality traits with each character by adopting procedure used in our previous studies [35], [36]. We started computing the Euclidean distance between the ratings for the self and the four political characters for each item (e.g. the square root of the squared difference of item 1 referred to self and item 1 referred to Berlusconi). We obtained a normalized dissimilarity score by summing the Euclidean distance of all the items and divided it for the maximum value (being 4 the maximum distance for each item, and having 25 items, we divided the sum for 100). This procedure allowed us to obtain a dissimilarity score between the voter and each politician. Dissimilarity scores of 1 and 0 indicate maximal difference and absence of difference, respectively. By subtracting the dissimilarity score from 1, we obtained the perceived similarity score which ranged from 0 (no similarity) to 1 (complete similarity). This score was entered in the correlation analyses.

In addition, we assessed the similarity between the perceived personality of each participant and of the four characters as ‘objectively’ assessed by averaging across the whole sample the ratings on each item.

## Results

### Ratings

Participants classified Di Pietro and Prodi as belonging to centre-left wing coalition (ratings 3.21 and 3.00, significantly lower than 4, *ts*<−3.3, *ps*<.01) and Berlusconi and Vespa as belonging to the center-right wing coalition (ratings were 5.75 and 4.73, significantly higher than 4, *ts*>3.34, *ps*<.01).

Media exposure ratings (where 1 = I know him through the media very well and 5 = I do not know him at all) were entered in a mixed model 2×4 ANOVA with group as between-subjects and character as within-subject factors. We found a main effect of character (F(3, 75) = 7.41, *p*<.001), but not of group (F(1, 25) = 1.13, *p*>.29). The interaction between group and character was not significant (F(3,75) = .04, *p*>.75). Duncan post-hoc comparisons showed that Berlusconi is considered more influent than anyone else (*ps*<.01). No other comparisons were significant.

Influence ratings (where 1 = very influent and 5 = not influent at all) were entered in a mixed model 2×4 ANOVA with group as between-subjects and character as within-subject factors. We found a main effect of character (F(3, 75) = 22.46, *p*<.001), but not of group (F(1, 25) = .004, *p*>.94). The interaction between group and character was not significant (F(3,75) = 1.07, *p*>.36). Duncan's post-hoc comparisons showed that Berlusconi is considered more influent than anyone else (mean 1.4, *ps*<.001). Moreover, Di Pietro (mean 2.6) was judged significantly more influent than Prodi (mean 3.6, *p*<.001), but only marginally significantly more influent than Vespa (mean 3.1, *p* = .05). Reports of positive minus negative emotions were used to compute an index of emotional positivity elicited by each character in each voter. These values were entered in a mixed model 2×4 ANOVA with group as between-subjects and character as within-subject factors. We found a main effect of group (F(1, 25) = 6.67, *p*<.05) which was accounted for by the less positive emotions reported by left-wing (−.93) than right-wing voters (−.17). The significance of the main effect of character (F(3, 75) = 3.84, *p*<.05) was explained by the more positive emotions elicited by Di Pietro (.56) with respect to the other characters (Berlusconi = −.77, Prodi = −.97, Vespa = −1.03; *ps*<.05). Duncan's post-hoc comparisons suggested that the significance of the group by character interaction (F(3, 75) = 19.37, *p*<.001) can be explained by an emotional ingroup bias. Indeed, significantly higher positive and negative emotion ratings were given to ingroup and outgroup characters respectively (all *ps*<.05, see Table 1), with the exception of Di Pietro who did not differ from Vespa within the right wing group (*p*>.5). Importantly, within the right-wing voters, emotions toward Di Pietro and Vespa characters did not differ significantly from zero (*ps*>.4). Within the left-wing group, emotions towards Prodi did not differ from 0 (*p*>.65), but were significantly more positive than emotions toward the outgroup characters (*ps*<.001). For each character, the emotions toward him differed significantly between the two groups (*ps*<.05).

**Table 1:** Mean differential emotion (mean positive emotion minus mean negative emotions) ratings for each character in each group (±SD).

	Di Pietro	Prodi	Berlusconi	Vespa
**Right-wing**	−0.2(2.2)	−2.2(2.0)	1.5(2.0)	0.2(1.2)
**Left-wing**	1.4(1.9)	0.3(2.3)	−3.1(1.4)	−2.3(1.8)

Negative scores indicate that negative emotions are predominant. Thus, a clear ingroup bias can be seen in both voters' groups.

### Saccadic Reaction Times

To have an index of interference, we computed the interference index for RTs by subtracting the mean RT in incongruent trials from the mean RT in congruent trials for each distracter condition and each subject. No subject had a score above or below 3 standard deviations from the mean of the group in any condition.

We entered this interference index in a mixed ANOVA with subjects political orientation (rightwing, leftwing) as a between factor and distracter (Di Pietro, Prodi, Berlusconi and Vespa) as within factor. We did not find any main effect, nor interactions (*ps*>.24, partial η^2^<.05). Means of the two way interactions are shown in Table 2.

**Table 2:** Reaction times.

	Di Pietro (ms)	Prodi (ms)	Berlusconi (ms)	Vespa (ms)
**Right-wing**	32.9 (17.9)	18.2 (23.7)	35.9 (24.8)	32.1(30.2)
**Left-wing**	39.7(25.4)	34.5(20.1)	35.3(23.1)	37.1(21.8)

Mean gaze cuing (incongruent minus congruent) in ms (±SD) effect for each condition in each group.

### Perceived similarity

We entered the subjective perceived similarity in a mixed ANOVA with subjects political orientation (rightwing, leftwing) as a between factor and distracter (Di Pietro, Prodi, Berlusconi and Vespa) as within factor. We did not find any main effect of the distractor (F(3, 75) = .66, *p* = .58). More importantly, we did not find any effect of the group, (F(1, 25) = .51, *p* = .48).

Not surprisingly, we found interaction between the participants orientation and the disctractors (F(3, 75) = 17.60, *p*<.001). Duncan's post hoc test showed that this interaction is accounted for by a more perceived similarity toward the ingroup than the outgroup characters. Indeed, left-wing voters perceived themselves more similar to Di Pietro and Prodi (mean perceived similarity: 0.77 and 0.73 respectively) than Berlusconi and Vespa (.60 and .62 respectively, *p*s<.01). Similarly, right-wing voters perceived themselves more similar to Berlusconi and Vespa (.74 and .72 respectively) than Di Pietro and Prodi (.61 and .56 respectively, *p*s<.01). Outgroup characters do not differ each other neither within the left-wing group, nor within the right-wing one (*p*s>.30).

### Saccadic Accuracy

**Figure 1:**
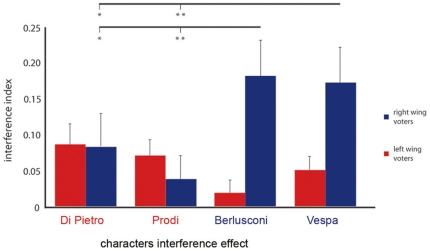
Interaction between participants political affiliation and characters interference effect. Influence of the political characters' gaze on the voters' oculomotor response. On the y axis is represented the interference index, deducted by the difference between the accuracy (percentage of correct responses) in congruent minus incongruent trials. Error bars represent Standard errors of mean (SEM). We reported significance (* = *p* <.05, ** = *p*<.01) only for the post-hoc comparisons between the characters' interference effect within the groups.

To have an index of interference, we subtracted the accuracy (percentage of correct responses) in incongruent trials from the accuracy in congruent trials from each condition and for each subject. We excluded a participant (left-wing, male) from the analysis because he scored above 3 standard deviations from the mean of the group in one condition. Interference index values were entered in a 2×4 mixed model ANOVA with voters group (centre-left, centre-right) as between-subjects factor and the distractor (the Di Pietro, Prodi, Vespa, Berlusconi character faces) as within-subjects factor. The ANOVA showed a trend towards a significant effect of group (F(1,25) = 3.79, *p* = .08), while the main effect of distractor was non significant (F(3, 75) = 1.53, *p* = .21). Importantly, the ANOVA showed that the crucial interaction between Distractor and Group was significant (F(3,75) = 6.87, *p*<.01, partial η^2^ = .18, see Figure 1). Since interference effect on accuracy in right wing participants was not distributed normally in one condition (Di Pietro, W = .83; *p*<.05) and the variance between the two groups was different in two conditions (Berlusconi and Vespa, Levenes's Fs(1, 25)>4.89 *p*s<.05) we used a bootstrapping resampling technique [37] to test our null hypothesis. We simulated 2.000 data sets of the same length of our original sample by randomly picking up with replacement the data from our original sample. So, in each simulation, we randomly assigned each data to each condition, entered the data in the same mixed model 2×4 ANOVA, computed the *F* for each main effect and for the interaction in order to build an *F* distribution from our original data. Finally, we computed the probability of the null hypothesis using these *F* distributions instead of the usual central F distribution. The *F* distributions that emerged after bootstrapping were very similar to the usual central *F* distribution ones and, consequently, we obtained very similar results. In particular, the main effect of the group still approached significance (*p* = .07), the main effect of the distractor was not significant (*p* = .26) and the crucial interaction was strongly significant (*p* = .001). As shown in Figure 1, Duncan's post-hoc test within groups revealed that Right-wing participants followed Berlusconi's gaze (accurate responses in congruent minus incongruent trials: 18.3%) more than Di Pietro's (8.7%, *p*<.05, Cohen's *d* = 0.57) and Prodi's (4%, *p*<.01, Cohen's *d* = 0.95) gaze. Furthermore, right wing voters followed Vespa's gaze (17.4%) more than Di Pietro's (*p*<.05, Cohen's *d* = 0.52) and Prodi's (*p*<.01, Cohen's *d* = 0.90) gaze. The interference effects were comparable for Berlusconi and Vespa (*p* = .77, Cohen's *d* = 0.05) and for Prodi and Di Pietro (*p* = .31, Cohen's *d* = 0.33). On the other side, Left-wing characters' gaze direction did not exert any significant influence on the oculomotor responses of left-wing participants (all *p*s>.14). Also, a difference between the two groups was found in the Berlusconi's gaze interference effect which was greater for right-wing participants than for the left-wing (2.1%, *p*<.005, Cohen's *d* = 1.20). Similarly, Vespa's gaze interference effect has found to be stronger in right-wing participants than in left-wing (5.2%, p<.05, Cohen's *d* = 0.91).

Previous behavioral studies demonstrated that longer or less accurate responses to incongruent than congruent trials are robustly and reliably induced by the interferential gaze of stranger models [11], [12], [13]. Using a series of one-sample t-tests, we assessed the strength of this effect by comparing the index of interference of effect each political character gaze against 0 (which means absence of interference) for each group. The interference of ingroup characters' gaze was significantly different from 0 in both left-wing (*ts*(13)>3.1; *ps*<.05) and right-wing participants (*ts*(12)>3.59, *ps*<.005).

Since Di Pietro interference effect was not normally distributed in the right-wing group (Shapiro-Wilk's W = .83; *p*<.05), we bootstrapped this difference data in the 13 right-wing participants 2000 times. So, we computed the mean difference (8.3%) of these 2000 samples and its confidence interval (CI, +95% = 19.9%; −95% = 2.2%). The lower bound of the CI does not include 0. Therefore, we can conclude that right-wing participants significantly follow the gaze of this out-group leader. For the other stimuli, being the data normally distributed (Ws>.88, *ps*>.07), we performed one-sample t-tests against zero.

Interestingly, unlike what reported with stranger gaze characters where interference is quite a robust phenomenon [11], [12], [13], some outgroup characters's gaze did not induce significant gaze following effects in right-wing voters (*p* = .24, for Prodi). On the other hand a lack of significant gaze following was found for Berlusconi's gaze left-wing voters (*t*(13) = 1.24; *p* = .23).

We ran the same analysis on the interference effect on RTs. In this case, all the characters induced a significant interference effect in both groups (*ts*>2.76, *ps*<.05).

### Accuracy interference index and ratings

No significant correlation between the interferential effect of characters' gaze and their reported influence or mediatic exposure was found (*ps*>.05).

### Accuracy interference index and perceived similarity in personality

We explored whether the interference effect of each character on the participants' oculomotor behavior can be at least partially explained by the perceived personality similarity between participant and character. To this aim we correlated the interference effect scores with the scores indexing the perceived similarity between the personality of the participants and that of each of the characters.

**Figure 2:**
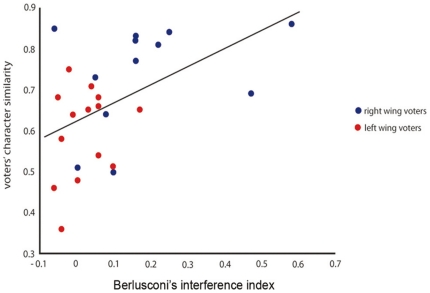
Correlation between participants perceived personality similarity with Berlusconi and his interference effect. On the y axis, the similarity scores, ranging from 0 (not similar at all) to 1 (completely similar) computed as described in the Methods. On the x axis, the interference index deducted by the difference between the accuracy (percentage of correct responses) in congruent minus incongruent trials.

We found a positive, significant correlation between the voters' perceived similarity with Berlusconi and the attracting influence of his gaze on their oculomotor behaviour (r = .50, *p*<.01; Figure 2). To be sure that the above positive correlation was not driven by outliers, we removed two subjects whose standardized residuals were above 2.5. The correlation became slightly stronger (r = .52; *p*<.01).

Perceived similarity in personality failed to correlate with the interference effect induced by the other characters (*rs*<.26, *ps*>.17). Because most of the correlated variables (6 out of 8) were not distributed normally (Ws<92; *ps*<.05), we ran the correlation analyses also on bootstrapped samples. 2000 resampled data sets were created and correlated. This procedure allowed us to check whether the confidence intervals of the Pearson's *rs* from the resampled data included zero. We found that the Pearson's *r* CI for the correlation between Berlusconi's interference effect and the perceived similarity with him did not include zero (+95% = .70, −95% = .15). Therefore, this correlation is to be considered significant. Importantly, all the other bootstrapped correlations between the character interference effect and the perceived similarity, included zero (CIs +95%<.55, −95%<−.02). Moreover, we aggregated across the whole sample the ratings concerning the perceived personality similarity and correlated this more ‘objective’ index with gaze interference. No correlation turned out to be significant (*r*s<.24, *p*>.22).

## Discussion

Gaze following behavior has shown to be an automatic behavior, supposedly impervious to highly complex variables such as political affiliation. Studies indicate that emotional cues in the face of a model can affect gaze cuing in human onlookers ([38], [39], but see [40]). Recent studies in monkeys [10] showed that social status of an individual within the group modulates gaze following of other members. However, little is known about whether this modulation can be due to dominance cues in the observed face or to social knowledge [41].

We anticipated that reflexive gaze following in humans might be influenced by highly complex cognitive and social dimensions as politic affiliation and personality dimensions linked to political ideology [25]. In this study, we investigated the interaction between the political affiliation of onlookers and distractors in an oculomotor task where the gaze direction of political characters could be spatially congruent or incongruent with the instruction to make directional saccades given to ingroup or outgroup electors. Also, since previous studies show the role of status in gaze following in monkey [10] and of familiarity on a gaze-cuing task in humans [41], we used characters who differed in terms of perceived influence in the Italian political landscape and in media exposure.

Accuracy results seem to suggest that, in each participant group, one of outgroup leaders did not exert a significant gaze following behavior. Specifically, right-wing voters seem not to be influenced by the gaze of Romano Prodi, a former centre-left Prime minister. In a similar vein, left-wing voters seem not to be interfered by the outgroup leader and actual Prime Minister, Silvio Berlusconi. This pattern of results may suggest that an active suppression on the gaze of some outgroup leaders is implemented. However, RTs analysis shows in both left-wing and right-wing voters a significant interference effect compared to zero for all the characters. Thus, although attractive, the hypothesis of an active suppression of attracting power of ougroup leaders gaze is based on a null result and involves only accuracy. Future studies on this issue are needed.

More importantly, we found that the stronger catching power of the ingroup political character gaze on voters occurred only in the right-wing voters, who were influenced by Berlusconi and Vespa more than by Di Pietro and Prodi. Even though these two characters have been judged as having a different media exposure and power in the political landscape (not surprisingly, since Berlusconi is the leader of the centre-right coalition and Prime minister in charge at the time of the experiment), they do not differ each other in their gaze interference power, suggesting that the result might have to deal more with the group affiliation than with the status. By contrast, no significant effects of in-group political characters' gaze were found in left-wing voters.

A possible explanation of the difference between left-wing and right-wing voters may involve personality differences in ingroup loyalty [31]. Indeed, conservatives are found to be more loyal to their group. Furthermore, conservatives are thought to be more sensitive to authoritarian figures and rely more on authority acceptance [27], [31]. It is thus possible that they follow the ruling group, more than simply the group they feel affiliated to. Since, at the time of data collection the centre-right group was fundamentally ruling the country, this alternative explanation cannot be disregarded. Future studies in a changed political situation or in different countries may help to better address this issue.

That left wing-voters lack of gaze-following behavior just with Berlusconi may be consistent with studies [42] showing that Italian left-wing voters detest the right-wing leader. Finally, the gaze interference effect exerted by the right wing leader was correlated to the voters' perceived similarity, in keeping with the evidence that Berlusconi is the leader that mostly capitalized on the personalization of politics strategy that has characterized several modern democracy systems in recent years [43].

Previous behavioural and neural studies of politics mainly focused on the dispositions of the participants [29], [30]. It has been shown for example that conservatorism but not liberalism is associated to the number of errors in tasks where a prepotent response has to be inhibited. This better behavioural performance of liberals in response conflict monitoring paralleled an higher sensitivity to response conflicts as indexed by the amplitude of No-Go N2 and Early Related Negativity Event Related potentials [29].

Using a gaze cuing paradigm in which the distractor was a schematic face, it has also been demonstrated that liberals exhibit a very large gaze cuing effect compared to conservatives [30]. Authors interpret these data arguing that while conservative ideology relies more on individuals, liberals are more likely to attend to social cues. These studies shed light on the notion of ideology as motivated social cognition [26], and try to link cognitive styles to Political orientation. Although interesting, the above studies do not address the important issue of how fundamental social behaviours like gaze following are modulated by Political affiliation in the interaction with members of a different vs. same political group.

In conclusion, unlike studies that investigated the behavioural and neural correlates affected by political variables by focusing on the dispositions of the participants, we demonstrate that a sophisticated blend of situational and dispositional factors underlies the capture of reflexive gaze following exerted on voters by the gaze of politicians. Future studies on the plasticity of this effect may provide new insights in the fundamental aspect of the human tendency to coalesce in large groups and complex societies.
